# The effect of child marriage on the utilization of maternal health care in Nepal: A cross-sectional analysis of Demographic and Health Survey 2016

**DOI:** 10.1371/journal.pone.0222643

**Published:** 2019-09-19

**Authors:** Kazutaka Sekine, Daniel J. Carter

**Affiliations:** 1 United Nation Population Fund Myanmar, Yangon, Myanmar; 2 Department of Infectious Disease Epidemiology, London School of Hygiene and Tropical Medicine, London, United Kingdom; African Population and Health Research Center, KENYA

## Abstract

A range of demographic and socioeconomic factors are known to account for enormous disparities in the uptake of maternal health care in low- and middle-income countries. In contrast, contextual factors such as child marriage are far less explored as a deterrent to the uptake of maternal health care. The present study aimed to assess the total effect of child marriage on the utilization of maternal health services in Nepal. This study drew on data from the Nepal Demographic and Health Survey 2016. The study restricted its analysis to a subsample of 3,970 currently married women of reproductive age who had at least one live birth in the five years preceding the survey. After descriptive analysis, logistic regression models were constructed to estimate adjusted odds ratios. The results of logistic regression controlling for confounders suggested child marriage decreased the likelihood of antenatal care visits (AOR 0.74; 95% CI 0.63–0.86), skilled attendance at delivery (AOR 0.66; 95% CI 0.56–0.78), facility-based delivery (AOR 0.65; 95% CI 0.56–0.77), and postnatal care use (AOR 0.80; 95% CI 0.67–0.96). The findings of this study reinforced the existing evidence for the adverse effect of child marriage on maternal health-seeking behaviors. Women’s restricted access to household resources, limited autonomy in decision-making, social isolation, and the dominant power of husbands and mothers-in-law may play a role in the findings. Addressing women’s social vulnerability as a barrier to accessing health care may help to increase the uptake of maternal health services.

## Introduction

Globally, around 303,000 women die annually from complications during pregnancy, childbirth, or the postnatal period. Almost all of these deaths (99%) occur in low- and middle-income countries [1]. Adolescents face a high risk of complications during pregnancy and childbirth due to their biological immaturity and socioeconomic factors [2]. A cross-sectional study from 29 countries found a heightened risk of eclampsia, puerperal endometritis, and systemic infection among adolescent mothers compared with mothers aged 20–24 years [3]. A study from 144 countries provided an estimate of a higher maternal mortality ratio of 260 per 100,000 live births for adolescent mothers, compared to the ratio of 190 for women aged 20–24 years [4].

A vast majority of these deaths are preventable if women receive adequate and timely medical care during pregnancy, childbirth, and the postpartum period [5]. It is widely held that the timely provision of quality obstetric care in facilities with adequate resources and staffing can save lives [6–8]. Skilled attendance at birth is an essential intervention for reducing the risk of adverse maternal and neonatal outcomes by preventing major complications including obstructed labor, eclampsia, puerperal sepsis, and obstetric hemorrhage [9]. It ensures the attendance of health professionals with the skills to provide medical care for normal and complicated deliveries. Antenatal care is a core component of routine maternal health services. Quality, comprehensive antenatal care can prevent and detect antenatal complications, such as anemia, malaria, pregnancy-induced hypertension, and preterm labor [10]. Antenatal care can also provide a valuable opportunity to encourage expectant mothers and families to deliver in a health facility and inform them about the danger signs for pregnancy complications [11].

There are enormous disparities in the uptake of maternal health care in low- and middle-income countries [12]. Previous research has suggested a range of demographic and socioeconomic factors account for these disparities. These factors include economic status, place of residence, geographic location, religion, women’s age, educational attainment, employment, and parity/birth order [13, 14]. Gabrysch and Campbell have highlighted sociocultural factors as a determinant for the delay in facility-based delivery in their framework [15]. Systematic reviews [13, 14] suggest, however, that sociocultural factors such as age at first marriage have been much less examined as a factor influencing maternal health-seeking behavior. In previous studies that identified factors influencing maternal health care use in Nepal, age at marriage was not taken into account [16–19]. Contextual factors such as child marriage deserve further exploration as a deterrent to the uptake of maternal health care.

Child marriage, defined as a formal marriage or informal union before the age of 18, is a stark reality for approximately 67 million young girls around the world [20]. While child marriage is illegal in most of the countries where the practice is prevalent, it is estimated that over 14 million girls get married as children every year [20]. In South Asia, 46 percent of women aged 20–24 were married or in union before the age of 18, which was higher than in any other region [20].

Although very little empirical research has examined the effect of child marriage on the uptake of maternal health care, women married as minors were found to be less likely to receive skilled care during pregnancy and at birth after controlling for sociodemographic indicators [21–24]. In Nepal, women who had married at the age of 15–17 made fewer prenatal visits than women who were married after the age of 17. Similarly, women married early were less likely to have skilled attendance at birth than those married at 18 or later [21]. In Pakistan, prenatal care by skilled medical care providers, skilled attendance at birth, and facility-based delivery were less common among women who were married as minors, compared to those who were married as adults [22]. In India, women who were married before the age of 18 were less likely to have their first delivery in a health facility than those who were married at 18 or older [23].

The evidence so far, however, is inconclusive. Previous research yielded mixed results for the effect of child marriage on maternal health services utilization across South Asian countries. For example, in India and Pakistan, age at first marriage did not appear to be correlated with the number of antenatal care visits [21, 22]. Similarly, no association between child marriage and skilled birth attendance was found in India [21]. Part of the reason for these results may be residual confounding or differing country context. Regression models of the previous studies omitted some relevant confounders such as religion, ethnic group, geographical area of residence, and husband’s education level, potentially resulting in inaccuracies in effect estimates. These variables have been shown to predict the utilization of maternal health services in Nepal [16–18]. The number of antenatal visits, which is predictive of the place of delivery [19, 25], was also omitted in adjusted models of the previous research [21–23]. Controlling for antenatal care use is critical as it provides a platform for educating pregnant women on the benefits of skilled attendance at delivery and postnatal care.

Although Nepal achieved the MDG 5 target of reducing the maternal mortality ratio by three quarters by 2015, its ratio remains high, at 258 per 100,000 live births [1]. The maternal mortality ratio among adolescent mothers is higher than that among women aged 20–29 years: 297 per 100,000 live births for adolescent mothers, 119 for women aged 20–24 years, and 191 for women aged 25–29 years [26]. One-third of pregnant women do not receive antenatal care at all or receive less than four antenatal care contacts [27]. Four in ten births are not attended by a skilled birth attendant [27].

Despite the fact that the legal age of female marriage in Nepal is 20, child marriage is pervasive. Although the prevalence of child marriage in Nepal had considerably decreased from 60% to 41% between 1996 to 2011 [28, 29], it has been stagnant around 40% after that period [30]. Married adolescent girls have little autonomy in decision making in their households. Approximately three quarters (73%) of them lack the ability to seek health care on their own and depend on their husband or mother-in-law to do so [27], which poses a barrier to utilization of maternal health care [31]. Child marriage may add a layer of vulnerability to married adolescent girls as it undermines women’s empowerment, deferring access to health care.

This study aimed to assess the total effect of child marriage on the utilization of maternal health services in Nepal. The study attempts to build an evidence base for a deeper understanding of the consequences of child marriage in the country and to inform maternal health policies.

## Methods

### Data

This study draws on data from the Nepal Demographic and Health Survey (NDHS) 2016, a cross-sectional study. It is one of the biggest household surveys conducted in Nepal and contains a rich set of data on reproductive health and demography. A nationally representative sample was obtained through the use of a two-stage, stratified random sampling design. The survey resulted in a sample of 12,862 women of reproductive age (15–49 years) and a response rate of 98% [30]. This study restricted its analysis to a subsample of 3,970 currently married women of reproductive age who had at least one live birth in the five years preceding the survey. For women with more than one live birth, only the most recent birth was analyzed to reduce recall bias and missing data. Restriction to married women was because childbirth mostly occurs within the confines of marriage in Nepal (30).

### Measures

#### Outcome variables

The outcome variables of interest are as follows:

- **Antenatal care** is defined as having a minimum of four antenatal contacts with a health care provider such as a doctor, nurse, or auxiliary nurse midwife during pregnancy. Although the World Health Organization (WHO) made a shift in its antenatal care guideline recommending a minimum of eight contacts [32], the Nepal government was promoting the traditional four-visit model at the time of the survey. This variable is coded as “1” if a woman falls under the definition and “0” if otherwise.
- **Skilled attendance at delivery** is defined as delivery in a health facility or at home with a skilled attendant such as a doctor, nurse, or auxiliary nurse midwife [33]. This definition of skilled birth attendants is in line with Nepal’s national safe motherhood and newborn health—long-term plan 2006–2017 [34]. This variable is coded as “1” if a woman falls under the definition and “0” if otherwise.
- **Facility-based delivery** is defined as delivery in a public or private health facility. This variable is coded as “1” if a woman falls under the definition and “0” if otherwise.
- **Postnatal care** is defined as receiving postnatal care from a skilled medical care provider in a health facility or at home at least once within 24 hours of birth. The timing is based on WHO recommendations [35]. This variable is coded as “1” if a woman falls under the definition and “0” if otherwise.

#### Exposure variable

The exposure variable of interest is child marriage. It is a dichotomous variable that is created based on self-reported age at first marriage. Women are coded as “1” if they are formally married or informally in union before the age of 18 and “0” if otherwise.

#### Control variables

**Fig 1:**
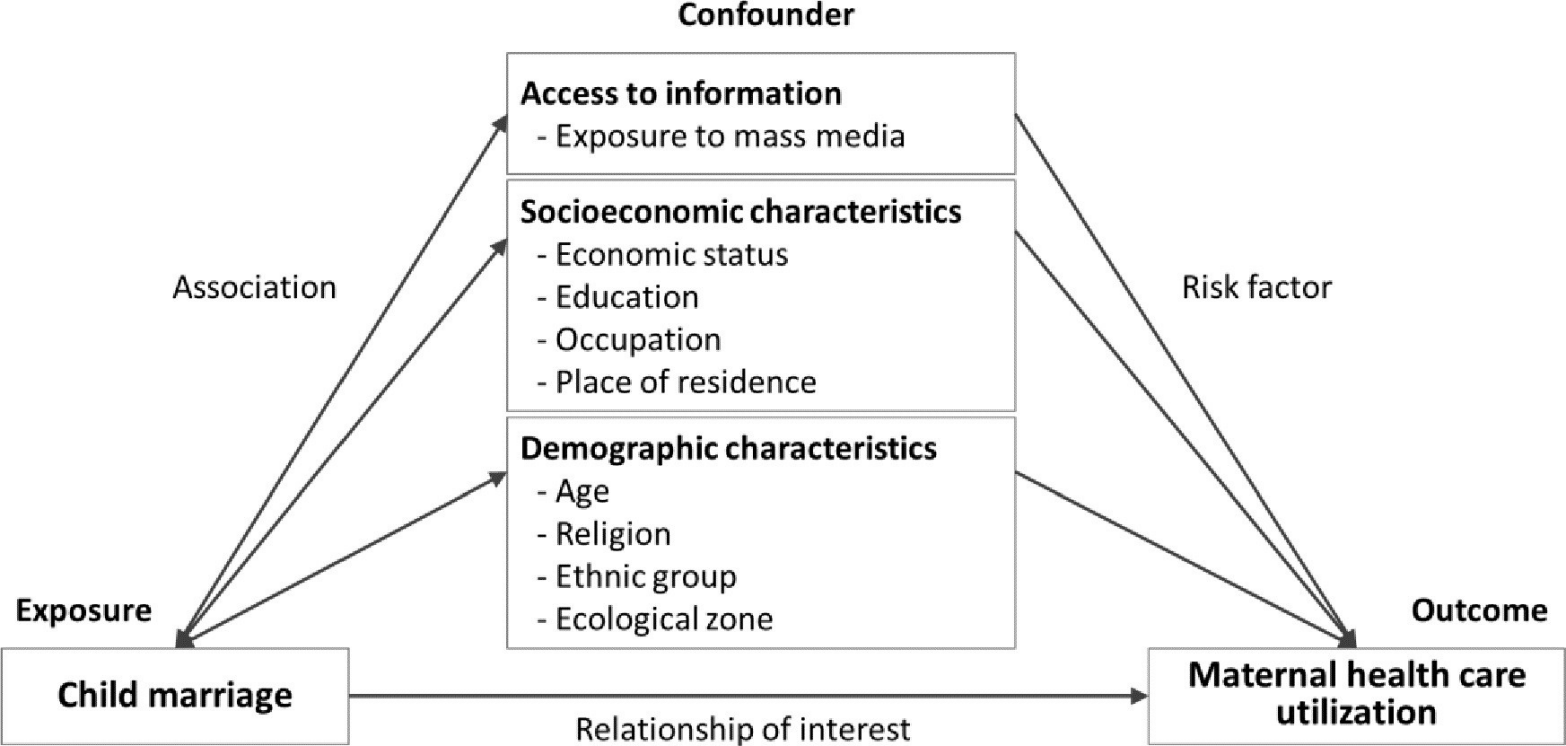
Framework for confounder selection.

In the literature, numerous variables have been shown to influence the incidence of child marriage and the uptake of maternal health care in Nepal [16–19, 30, 36]. We used background knowledge based on the literature review to develop a framework for the selection of confounders (Fig 1). A causal diagram was also created using DAGitty [37] to depict the hypothesis of causal relationships relevant to our research question and find a minimal sufficient adjustment set (S1 Appendix). It provides a visual, yet logically rigorous aid in confounder selection. It is simplification of reality, and thus cannot represent all causal relationships. The potential confounders included in regression analyses are presented in Table 1.

**Table 1:** Control variables and their measurements.

Control variable	Measurement scale
Age of the woman at interview	15–19, 20–24, 25–29, 30–34, or ≥35
Age of the husband at interview	15–19, 20–24, 25–29, 30–34, or ≥35
Religion	Hindu or non-Hindu
Ethnic group	Advantaged or disadvantaged
Ecological zone	Mountain, hill, or lowlands (also known as Terai)
Economic status of the household	Poor, middle income, or rich
Highest level of education that women have attained	No education, primary education, or secondary/higher education
Highest level of education that the husband has attained	No education, primary education, or secondary/higher education
Women’s job	No job/low skilled job or paid skilled jobs
Place of residence	Urban or rural
Exposure to mass media	‘no exposure to radio or television,’ ‘exposure to either radio or television at least once a week,’ ‘exposure to both radio and television at least once a week’

In the NDHS, the household wealth status was measured as a composite index comprised of housing construction materials and household assets. The wealth index factor score was allocated to each household based on the composite index. Households were divided equally into three groups: poor, middle income, and rich. Women’s job was also re-categorized into two groups. No job, agricultural job and unskilled manual jobs were grouped as “no job/low skilled job,” and professional, clerical or services job and skilled manual jobs were categorized as “paid skilled jobs.” Ethnic groups, such as Dalit and Janajati, and Muslims were categorized as “disadvantaged” and other groups as “advantaged.”

### Statistical analysis

Demographic and socioeconomic characteristics of the sample as well as the outcome variables were described using weighted percentages. The differentials of child marriage by demographic and socioeconomic indicators were examined using χ^2^ tests. Associations between child marriage and maternal care use were assessed by calculating odds ratios (ORs) with 95% confidence intervals (CIs) using logistic regression models. After calculating unadjusted ORs, logistic regression models were then constructed to estimate adjusted odds ratios (AORs) while controlling for confounders. In Model 1, specified for antenatal care visits, all the control variables mentioned above were controlled for. While keeping all the control variables included in Model 1, antenatal care use was additionally conftrolled for to follow the temporal sequence in Model 2 specified for skilled attendance at delivery and facility-based delivery. Both antenatal care use and skilled attendance at delivery were added in Model, 3 specified for postnatal care.

All the data analyses were weighted to account for the complexities in the sample design, such as stratified sampling and probabilities of unequal sample selection between regions. Using the national women’s weighting for the entire NDHS allows for analyses that produce nationally representative estimates. All statistical analyses for this study were performed using Stata 13 (StataCorp LP, College Station, TX).

### Ethical statement

The study used a de-identified secondary dataset of the NDHS 2016, which is freely and publicly available. The standard protocols, data collection tools and procedures of the NDHS were approved by the Nepal Health Research Council and ICF Macro Institutional Review Board in Calverton, Maryland, USA [30]. Written informed consent to carry out the interview was obtained from the household head. Ethical approval was obtained from the London School of Hygiene and Tropical Medicine to conduct this study.

## Results

### Sample characteristics

The NDHS 2016 dataset generated a sample of 3,970 currently married women aged 15–49 years who had a live birth in the past five years. In this group, the prevalence of child marriage was 53%. The majority of the participants were in their 20s and had a husband aged between 25 and 49 years (Table 2). Most of them were Hindu, had no job or a low skilled job, and had one or two live births. Approximately half of them were from lowlands, resided in urban areas, belonged to advantaged ethnic groups, and attained a secondary or higher level of education. Regular exposure to mass media was rare.

**Table 2:** Demographic and socioeconomic characteristics of currently married Nepali women aged 15–49 who had a live birth in the past five years.

Variable		Participants						P-value
		Total (n = 3,970)		Child marriage (n = 2,138)		Adult marriage (n = 1,832)		
		n	%	n	%	n	%	
Age of women at interview								P<0.001
	15–19	341	8.3	320	14.7	21	1.1	
	20–24	1,301	31.8	756	35.3	545	27.9	
	25–29	1,337	34.6	612	29.1	725	40.9	
	30–34	644	16.3	299	13.7	345	19.1	
	≥35	347	9.0	151	7.2	196	10.9	
Age of husbands at interview								P<0.001
	15–19	49	1.1	31	1.4	18	0.8	
	20–24	676	15.5	438	18.9	238	11.6	
	25–29	1,210	30.3	643	31.0	567	29.4	
	30–34	1,092	28.3	547	26.0	545	30.9	
	≥35	943	24.9	479	22.8	464	27.3	
Religion								P = 0.857
	Hindu	3,458	85.7	1,851	85.5	1,607	85.8	
	Others	512	14.3	287	14.5	225	14.2	
Ethnic group								P = 0.052
	Advantaged	2,001	50.9	1,034	48.7	967	53.3	
	Disadvantaged	1,969	49.2	1,104	51.3	865	46.7	
Ecological zone								P<0.001
	Mountain	323	6.7	177	6.7	146	6.7	
	Hill	1,724	40.2	859	33.3	865	48.0	
	Lowland	1,923	53.1	1,102	60.0	821	45.3	
Economic status								P<0.001
	Poor	1,323	27.9	780	30.9	543	24.5	
	Middle	1,324	34.5	775	39.1	549	29.3	
	Rich	1,323	37.6	583	30.0	740	46.3	
Highest level of women's education								P<0.001
	No education	1,218	31.5	802	39.5	416	22.3	
	Primary	755	19.4	497	24.6	258	13.5	
	Secondary or higher	1,997	49.2	839	35.9	1,158	64.2	
Highest level of husband's education								P<0.001
	No education	504	13.7	364	19.0	140	7.6	
	Primary	840	21.4	542	26.4	298	15.7	
	Secondary or higher	2,621	64.9	1,229	54.5	1,392	76.7	
Women's job								P<0.001
	No job or low skilled job	3,477	86.5	1,943	90.9	1,534	81.6	
	Paid skilled jobs	493.0	13.47	195	9.1	298	18.4	
Place of residence								P<0.001
	Urban	2,314	55.5	1,157	48.5	1,157	63.5	
	Rural	1,656	44.5	981	51.5	675	36.5	
Exposure to mass media								P<0.001
	No exposure to radio or television	2,740	69.7	1,620	77.0	1,120	61.3	
	Exposure to either radio or television at least once a week	757	18.3	327	13.6	430	23.5	
	Exposure to both radio and television at least once a week	473	12.1	191	9.4	282	15.2	

% = weighted percentage Absolute number of participants does not perfectly correspond to percentages presented because weighted analyses were used. P-value is for the differentials of child marriage by demographic and socioeconomic characteristics.

### Differentials of child marriage by demographic and socioeconomic characteristics

Among the sample, there were remarkable differences in the prevalence of child marriage by demographic and socioeconomic characteristics. The proportion of child marriage was higher among women who were young, poor, less educated, living in lowlands or rural areas, who belonged to disadvantaged ethnic groups and had no job or a low skilled job. Women married as children tended to have younger and less educated husbands, but were less exposed to mass media (Table 2).

### Utilization of maternal health care

Among the sample, the receipt of all maternal health care exceeded 50% (Table 3). Four or more antenatal contacts with health care providers were accomplished among 63% of the sample. Skilled attendance at delivery and facility-based delivery was found in 61% and 58% of the sample, respectively. Postnatal contacts within 24 hours of birth were made among 52% of the sample. Women married as minors had lower uptake of all the maternal health services of interest.

**Table 3:** Associations between child marriage and maternal care use among currently married Nepali women aged 15–49 years who had a live birth in the past five years (n = 3,970).

Outcome variables		Overall		Child marriage (n = 2,138)		Adult marriage (n = 1,832)		OR (95% CI)	Model 1: Adjusted OR (95% CI)^a^	Model 2: Adjusted OR (95% CI)^b^	Model 3: Adjusted OR (95% CI)^c^
		n (weighted %)		n (weighted %)		n (weighted %)					
Four or more antenatal care contacts								0.53 (0.47–0.61)***	0.74 (0.63–0.86)***		
	Yes	2,534	(63.0)	1,223	(54.9)	1,311	(72.1)				
	No	1,436	(37.0)	915	(45.1)	521	(27.9)				
Skilled attendance at delivery								0.49 (0.43–0.56)***		0.66 (0.56–0.78)***	
	Yes	2,421	(61.0)	1,141	(51.8)	1,280	(71.4)				
	No	1,549	(39.0)	997	(48.2)	552	(28.6)				
Facility-based delivery								0.47 (0.41–0.54)***		0.65 (0.56–0.77)***	
	Yes	2,310	(57.5)	1,066	(47.4)	1,244	(69.0)				
	No	1,660	(42.5)	1,072	(52.6)	588	(31.0)				
Postnatal care within 24 hours of childbirth								0.52 (0.45–0.59)***			0.80 (0.67–0.96)*
	Yes	2,054	(51.7)	945	(42.8)	1,109	(62.0)				
	No	1,916	(48.3)	1,193	(57.3)	723	(38.0)				

OR = odds ratio; CI = confidence interval;

*** p<0.001;

** p<0.01;

* p<0.05

^a^ Analysis adjusted for age of the woman and her husband, religion, ethnic group, ecological zone, economic status, the highest level of education attained by the woman and the husband, women’s job, place of residence, and exposure to mass media.

^b^ Analysis adjusted for antenatal care visits in addition to all the variables adjusted in Model 1.

^c^ Analysis adjusted for skilled attendance at delivery in addition to all the variables adjusted in Model 2.

### Associations between child marriage and maternal care use

In unadjusted analyses, child marriage was significantly negatively associated with all outcome variables (Table 3). Women married as children were less likely to make a minimum of four antenatal care contacts with a health care provider (OR 0.53; 95% CI 0.47–0.61), have skilled attendance at delivery (OR 0.49; 95% CI 0.43–0.56), give birth in a health facility (OR 0.47; 95% CI 0.41–0.54), and receive postnatal care from a skilled medical care provider in a health facility or at home within 24 hours of birth (OR 0.52; 95% CI 0.45–0.59). The effect of child marriage remained strong after controlling for confounders (Table 3). Regression analysis of Model 1 showed that women married as children were 26% less likely to utilize antenatal care (AOR 0.74; 95% CI 0.63–0.86). Regression analysis of Model 2, additionally adjusted for antenatal care, suggested that child marriage decreased the likelihood of skilled attendance at birth and facility-based delivery by 34% (AOR 0.66; 95% CI 0.56–0.78) and 35% (AOR 0.65; 95% CI 0.56–0.77), respectively. After controlling for skilled attendance at delivery in Model 3, women married as children were found to be 20% less likely to have received postnatal care (AOR 0.80; 95% CI 0.67–0.96). The effect sizes estimated in the series of regression analyses were similar across the four outcomes.

## Discussion

The present study revealed that among married women in Nepal with at least one live birth in the past five years, child marriage decreased the odds of using antenatal care, skilled attendance at delivery, facility-based delivery, and postnatal care. The associations remained strong after adjusting for social inequality markers (including poverty, rural residence, no formal education, belonging to disadvantaged ethnic groups) and other confounders in regression models. The lower level of maternal health services utilization persisted throughout women’s reproductive life. These findings indicate that child marriage hinders women’s access to maternal health care; therefore, women who are married young may be at a heightened risk of maternal mortality and morbidity as their complications during pregnancy and childbirth are less likely to be prevented and treated in a timely way.

The findings in this study reinforce existing evidence for the adverse consequences of child marriage on maternal health-seeking behaviors [21–24]. Effect sizes are not directly comparable because of differences in the definitions of outcome variables. What stands out is that the notable effect of child marriage was found consistently across all of the outcome indicators. To our knowledge, this study is the first study to yield an estimated effect of child marriage on the use of postpartum care. In previous research [21–24], analyses were restricted to young cohorts of women aged 15–24 or 20–24. In contrast, the present study included all married women regardless of age, leading to results which are more representative of the situation in Nepal. Using a broader age range for the sample resulted in a much larger sample size, relative to the previous studies, and provided enhanced confidence in estimates while focusing on recent effects. In addition, greater inclusion of confounders in adjusted models may have led to increased accuracy in estimation.

An intertwined complex network of social and economic constraints facing women may be at play in the relationships between child marriage and maternal health-seeking behaviors in Nepal. Given the patriarchal structure of Nepalese society, husbands have dominant power within households, and gender relations are persistent [38]. Women have restricted access to household resources, limited autonomy in decision-making regarding their health care, and also require the husband’s permission to go out. Such women’s status may affect the economic and social accessibility of maternal health care. In fact, a higher percentage of young mothers aged below 20 years reported disapproval for delivering in a health facility from their husband or family, compared to adult mothers [30]. Five or more years of spousal age difference was observed among 40% of the sample. Women’s young age relative to their husbands may put women under the control of husbands and exacerbate their inability to seek health care. In addition, traditional marital living arrangements in Nepal involve residing with husband’s parents and extended families. When a newly married woman moves into her husband’ home in South Asia, it is customary for her to come under the control of her mother-in-law and elder sisters-in-law. Some of mothers-in-law often have historically had a negative influence on the health-seeking behavior of their daughters-in-law because of their own past experience where they perceived no benefit from antenatal care [39]. Mothers-in-law also have control over household resources, which prevents pregnant women from accessing antenatal care [39]. Past research in India found that women in joint households were less likely to use maternal health care services than women in nuclear households [40]. Women’s access to household resources, limited autonomy in decision-making, and movement restrictions may be on the causal pathway between child marriage and maternal care use in Nepal. Previous studies in Nepal found associations between higher level of women’s autonomy in decision-making and uptake of maternal health services [31, 41]. An intricate web of sociocultural factors such as gender norms and intra-household gender relations may be an underlying cause for the negative association between child marriage and maternal health-seeking behaviors. In-depth research is needed to explore cultural and socioeconomic factors that impede women married as children in utilizing maternal health care and to elucidate pathways for how child marriage causes a lower level of maternal health care use. Research to address what works in mitigating the adverse effect of child marriage is also needed.

After marriage, due to domestic duties and control by husbands and mothers-in-law, some women married as children face physical and social isolation and are deprived of supportive and cohesive friendship networks [42]. Because of their limited social participation, child marriage may put women at a disadvantage in accessing information about the importance of maternal health care and danger signs during pregnancy and childbirth. This contributes to the first delay in the Three Delays Model by Thaddeus and Maine [43]. In addition, access to cash for transportation and medical care or requirements to have permission from the husband or mother-in-law may pose a major barrier after making decisions to seek maternal health care. This barrier leads to the second delay in the same model. Unmeasured confounders such as quality of care could partially explain the observed associations; thus, residual confounding effects may remain.

Women married as minors need to be recognized as vulnerable in Nepal’s national maternal health policy and strategies. Despite the national free delivery care policy where essential health care including antenatal care and facility-based delivery are free of charge across the country, child marriage hinders women’s access to these services. The findings underscore the need to challenge harmful gender norms that prevent women from accessing the services and perpetuate child marriage. Improving women’s autonomy in decision-making may help to increase the coverage of maternal health services. Increased community advocacy for changing social and gender norms is needed. Furthermore, the negative consequences of child marriage need to be highlighted in national strategies to end child marriage.

Apart from the possible residual confounding effects discussed above, the study has a few more limitations that should be considered. First, the NDHS data are prone to recall and social desirability biases because of the nature of self-reported data. This study considered only the most recent births in the past five years to reduce recall bias and missing data. The NDHS data collection team took measures to collect accurate data from interviewed women by ensuring privacy and confidentiality. These biases cut across all women regardless of the age of first marriage. Therefore, the effect estimates of this study are assumed to have a minimal bias. Secondly, as the analyses of the study were cross-sectional, the study cannot infer causality between child marriage and the outcomes of interest. Thirdly, constraints of supply side determining the availability of maternal care were not available in the dataset, therefore, were not included as control variable. Fourthly, it is possible that some of the confounders adjusted for are factors on the causal pathway between child marriage and health service utilization as there may be changes in socioeconomic status due to marriage. However, most marriages in Nepal are arranged based on the same or similar caste, religion and education level of brides and grooms. Therefore, a drastic change in socioeconomic status through marriage is unlikely. However, a minimal temporality bias can be assumed as marriage usually takes place before pregnancy in Nepal. Despite these limitations, the strength of the study is that it is based on nationally representative data, which were generated by the well-established national survey that used standardized measures and protocols. The findings are generalizable to married women aged 15–49 years across the country, and this study was able to address a range of sources of confounding effects.

## Conclusion

This study reveals that child marriage has a detrimental impact on the uptake of maternal health care in Nepal. This finding offers important insights into the consequences of child marriage and has implications for policymakers, planners, and health practitioners to consider. Maternal health and social development programs should address gender norms and intra-household gender relations that hinder women’s access to health care. Future research should explore the pathway through which child marriage causes a lower level of maternal health care use.

## Supporting information

S1 Appendix (DOCX)
