# Supplementary material for: The effect of child marriage on the utilization of maternal health care in Nepal: A cross-sectional analysis of Demographic and Health Survey 2016
Source: PLoS One. 2019 Sep 19;14(9):e0222643. doi: 10.1371/journal.pone.0222643 (PMC6752778; doi:10.1371/journal.pone.0222643)
Supplement: S1 Appendix — (DOCX) [file pone.0222643.s001.docx]

**S1 Appendix**

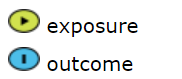

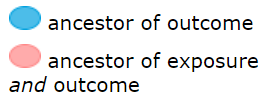

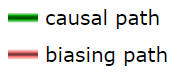


Note: Variables with a biasing path were adjusted for in multivariate logistic regressions in this study to estimate the total effect of child marriage.
